# Supplementary material for: Development of a targeted BioPROTAC degrader selective for misfolded SOD1
Source: Nat Commun. 2025 Nov 10;16:9713. doi: 10.1038/s41467-025-65481-w (PMC12603324; doi:10.1038/s41467-025-65481-w)
Supplement: Supplementary file 1 — Supplementary Information [file 41467_2025_65481_MOESM1_ESM.docx]

# Supplementary Information

# Development of a targeted BioPROTAC degrader selective for misfolded SOD1

Christen G. Chisholm^1^*, Rachael Bartlett^1^, Mikayla L. Brown^1^, Emma-Jayne Proctor^1^, Natalie E. Farrawell^1^, Jody Gorman^1^, Fabien Delerue^2,3^, Lars M. Ittner^2,4^, Kara L. Vine-Perrow^1^, Heath Ecroyd^1^, Neil R. Cashman^5,6^, Darren N. Saunders^7^, Luke McAlary^1^, Jeremy S. Lum^8^* and Justin J. Yerbury^1#^

#Deceased: Justin J. Yerbury

**Affiliations:**

^1^Molecular Horizons and School of Science, University of Wollongong, Wollongong, NSW, 2522, Australia

^2^Dementia Research Centre, Macquarie Medical School, Faculty of Medicine, Health and Human Sciences, Macquarie University, Sydney, NSW, 2109, Australia

^3^Department of Genetics, The University of Texas MD Anderson Cancer Centre, Houston, TX, 77030, USA.

^4^Celosia Therapeutics, Sydney, NSW, 2109, Australia

^5^Djavad Mowafaghian Centre for Brain Health, University of British Columbia, Vancouver, BC, V6T 1Z4, Canada

^6^ProMIS Neurosciences, Toronto, ON, M4S 3E2, Canada

^7^School of Medical Sciences, Faculty of Medicine and Health, University of Sydney, Sydney, NSW, 2050, Australia

^8^Molecular Horizons and School of Medical, Indigenous and Health Sciences, University of Wollongong, Wollongong, NSW, 2522, Australia

*Correspondence

jlum@uow.edu.au (J.S.L)

christen@uow.edu.au (C.G.C)

**
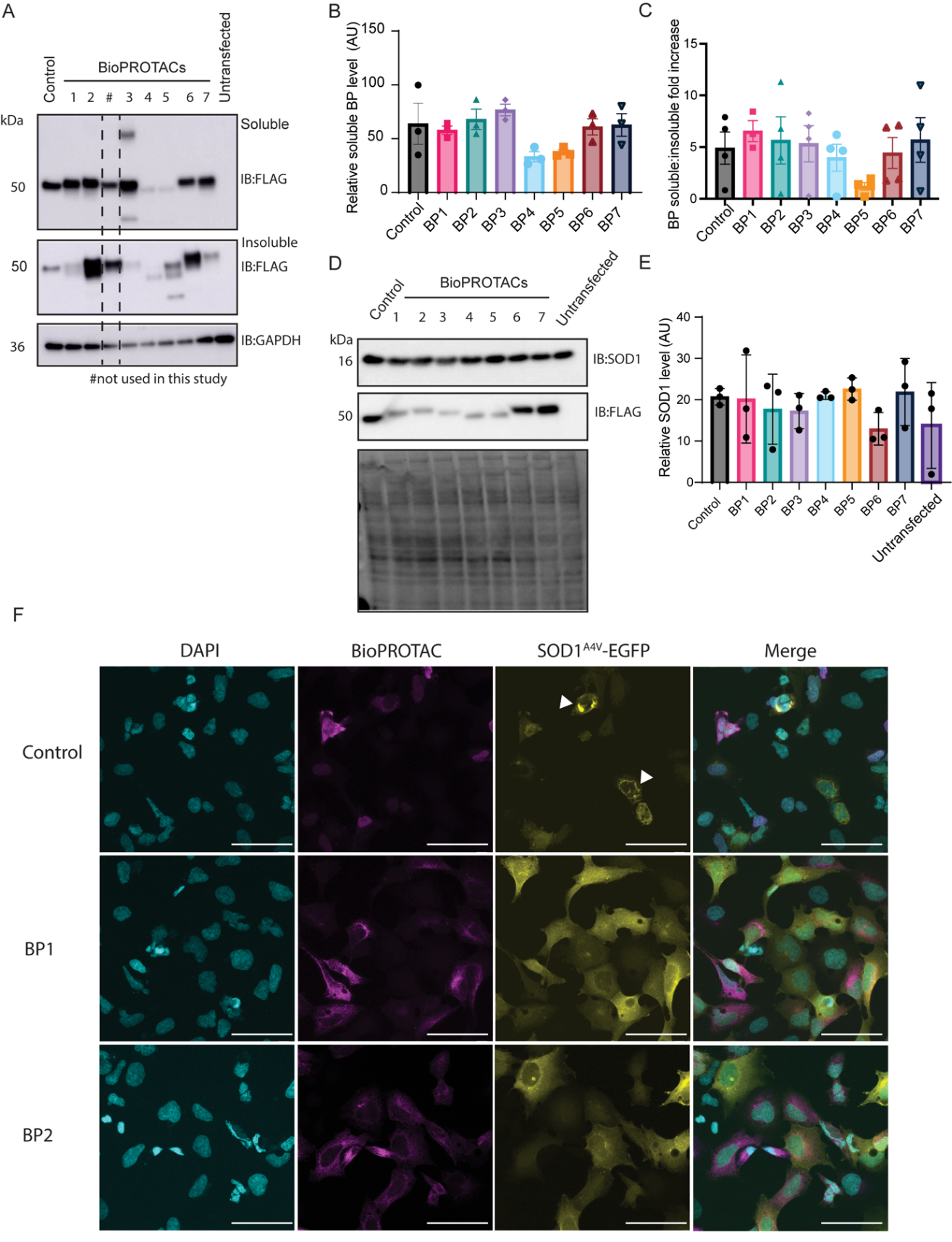
**

# **Supplementary Figure 1**

**. BioPROTACs are expressed in the soluble fraction of the nucleus and cytoplasm, and do not reduce endogenous SOD1. (A and B)** BioPROTAC expression was assessed in the soluble and insoluble fractions from HEK293 lysates. **(C)** Fold increase in expression of soluble to insoluble fraction was calculated for each BioPROTAC. **(D and E)** The soluble fraction was also assessed for SOD1 levels to determine effect on endogenous SOD1. **(F)** Immunocytochemistry was used to assess BioPROTAC expression levels. Representative images for BP1, BP2 and the control in HEK293 cells transiently transfected to express BioPROTACs (magenta) and SOD1^A4V^-EGFP (yellow). Scale bars represent 50 µm. White arrows show aggregates. For all graphs, bars represent mean ± SEM. Statistical significance was determined using **(B and C)** ordinary one-way ANOVA paired with Tukey’s multiple comparisons test or **(E)** repeated measures one-way ANOVA paired with Dunnett’s multiple comparisons test. All statistical values are shown in Supplementary Table 2. Blots are representative from at least 3 independent experiments. Raw data and exact P-values are shown in source data file.


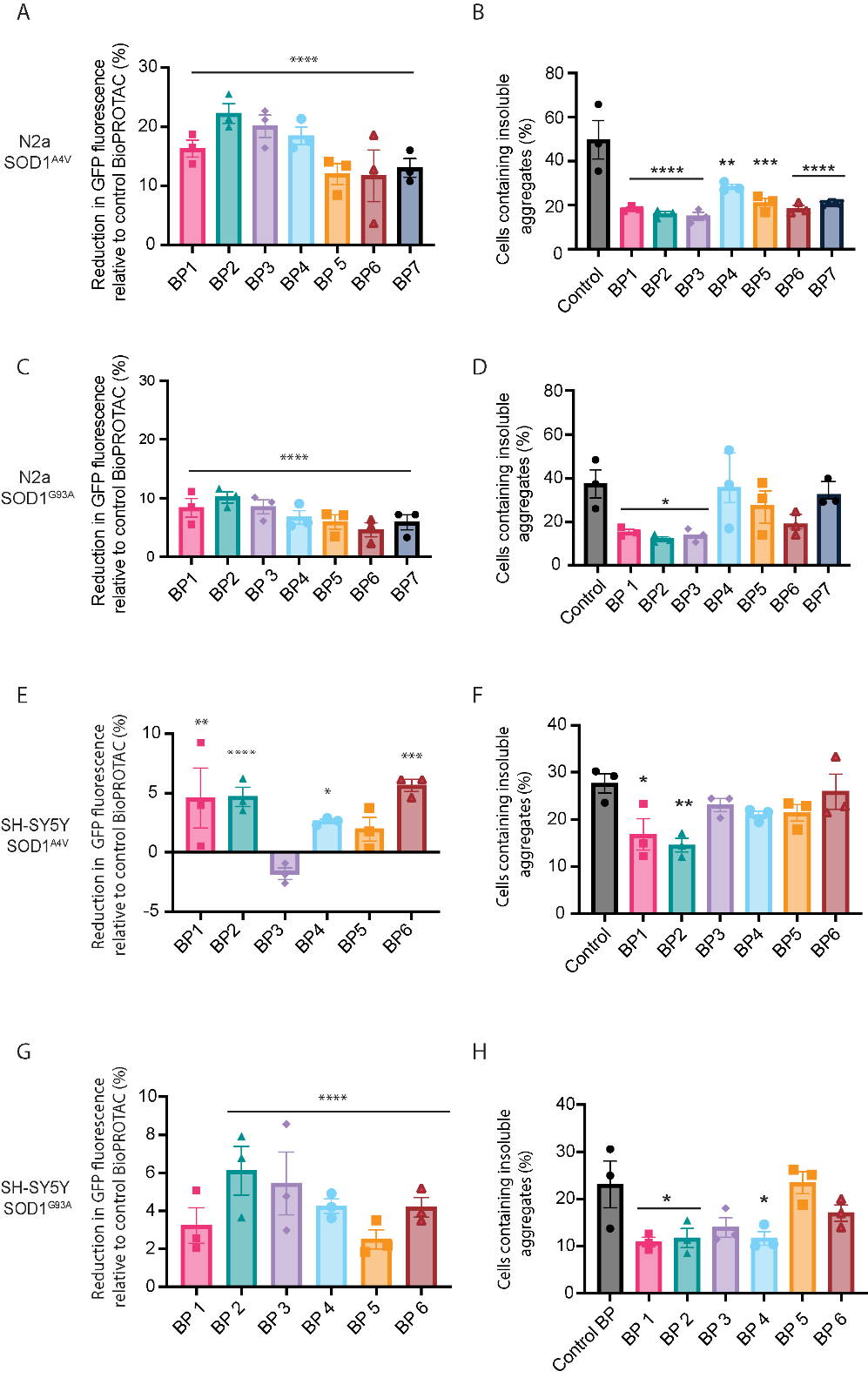


# Supplementary Figure 2

**. BioPROTACs degrade SOD1 variants in multiple cell types.** Total **(A)** SOD1^A4V^‑EGFP and **(C)** SOD1^G93A^-EGFP fluorescence in Neuro2A cells expressing BioPROTACs relative to cells co-transfected with the control over 48 hr. The number of cells containing insoluble **(B)** SOD1^A4V^‑EGFP and **(D)** SOD1^G93A^-EGFP aggregates in Neuro2A cells expressing BioPROTACs relative to cells co-transfected with the control was quantified using the saponin permeability assay. Total **(E)** SOD1^A4V^‑EGFP and **(G)** SOD1^G93A^-EGFP fluorescence in SH-SY5Y cells expressing BioPROTACs relative to cells co-transfected with the control over 48 hr. The number of cells containing insoluble **(F)** SOD1^A4V^‑EGFP and **(H)** SOD1^G93A^-EGFP aggregates in SH-SY5Y cells expressing BioPROTACs relative to cells co-transfected with the control was quantified using the saponin permeability assay. For all graphs, bars represent mean ± SEM (* *P* < 0.05, ** *P* < 0.01, *** *P* < 0.001, **** *P* < 0.0001). Statistical significance was determined using **(A, C and E-H)** repeated measures or **(B and D)** ordinary one-way ANOVA paired with Dunnett’s multiple comparisons test. Raw data and exact P-values are shown in source data file.


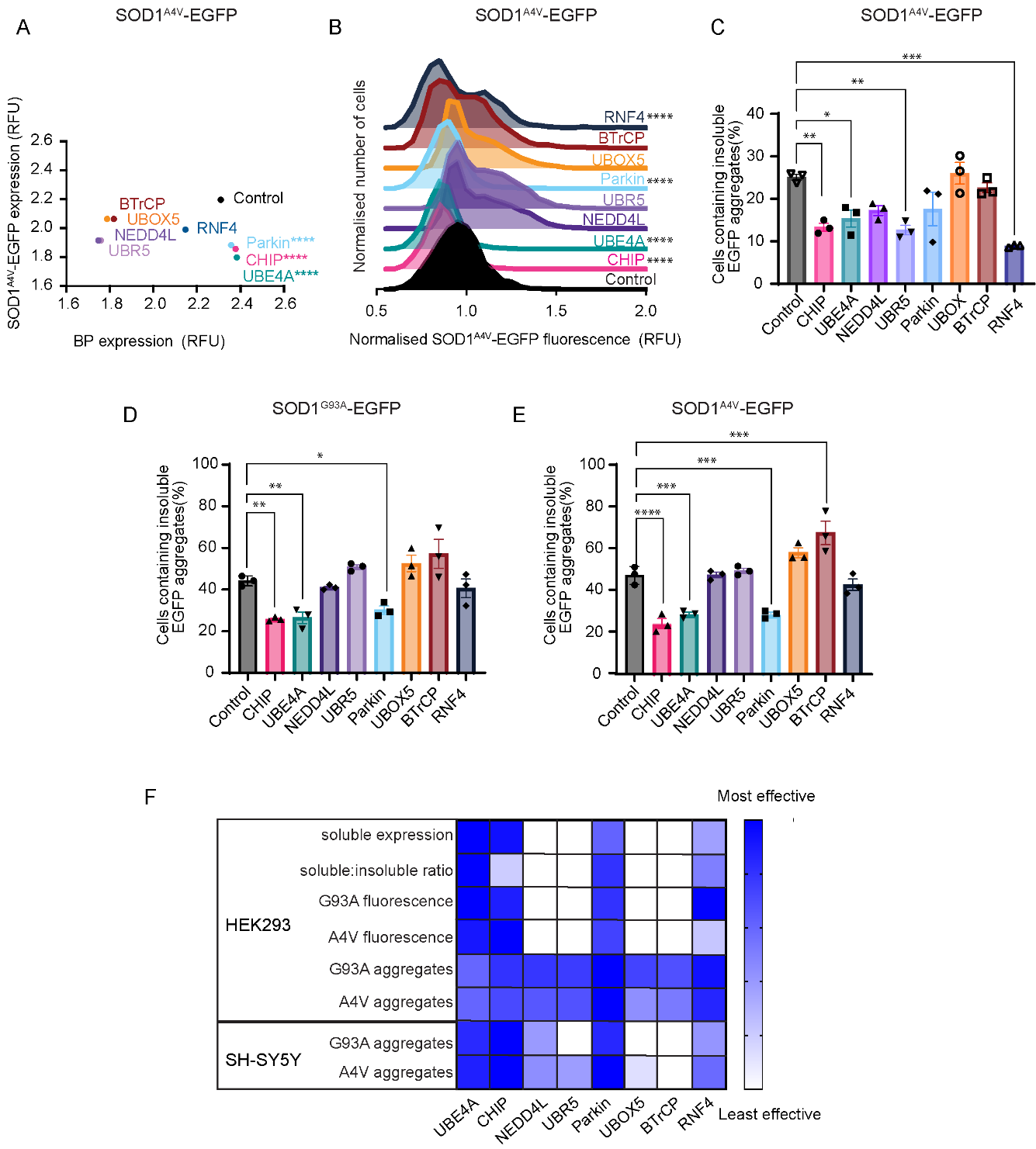


# Supplementary Figure 3

**.** **Reduction of misfolded SOD1 is ligase dependent and consistent across cell lines and SOD1 mutants. (A)** Immunocytochemistry was used to assess BioPROTAC and SOD1^A4V^‑EGFP expression in HEK293 cells. **(B)** The reduction in SOD1^A4V^‑EGFP fluorescence compared to control was determined when expression levels of the BioPROTACs were normalised. **(C)** The number of cells containing insoluble SOD1^A4V^‑EGFP aggregates in HEK293 cells expressing BioPROTACs relative to cells co-transfected with the control was quantified using the saponin permeability assay. **(D)** The number of cells containing insoluble SOD1^G93A^‑EGFP aggregates in SH-SY5Y cells expressing BioPROTACs relative to cells co-transfected with the control. **(E)** The number of cells containing insoluble SOD1^A4V^‑EGFP aggregates in SH-SY5Y cells expressing BioPROTACs relative to cells co-transfected with the control. **(F)** A weighted heat map comparing the relative efficacy of BioPROTACs in the various assays. For all graphs, bars represent mean ± SEM (* *P* < 0.05, ** *P* < 0.01, *** *P* < 0.001, **** *P* < 0.0001). Statistical significance was determined using **(A and B)** Kruskal-Wallis one-way ANOVA paired with Dunn’s multiple comparisons test or **(C-E)** ordinary one-way ANOVA paired with Dunnett’s multiple comparisons test. Raw data and exact P-values are shown in source data file and Supplementary Table 2.


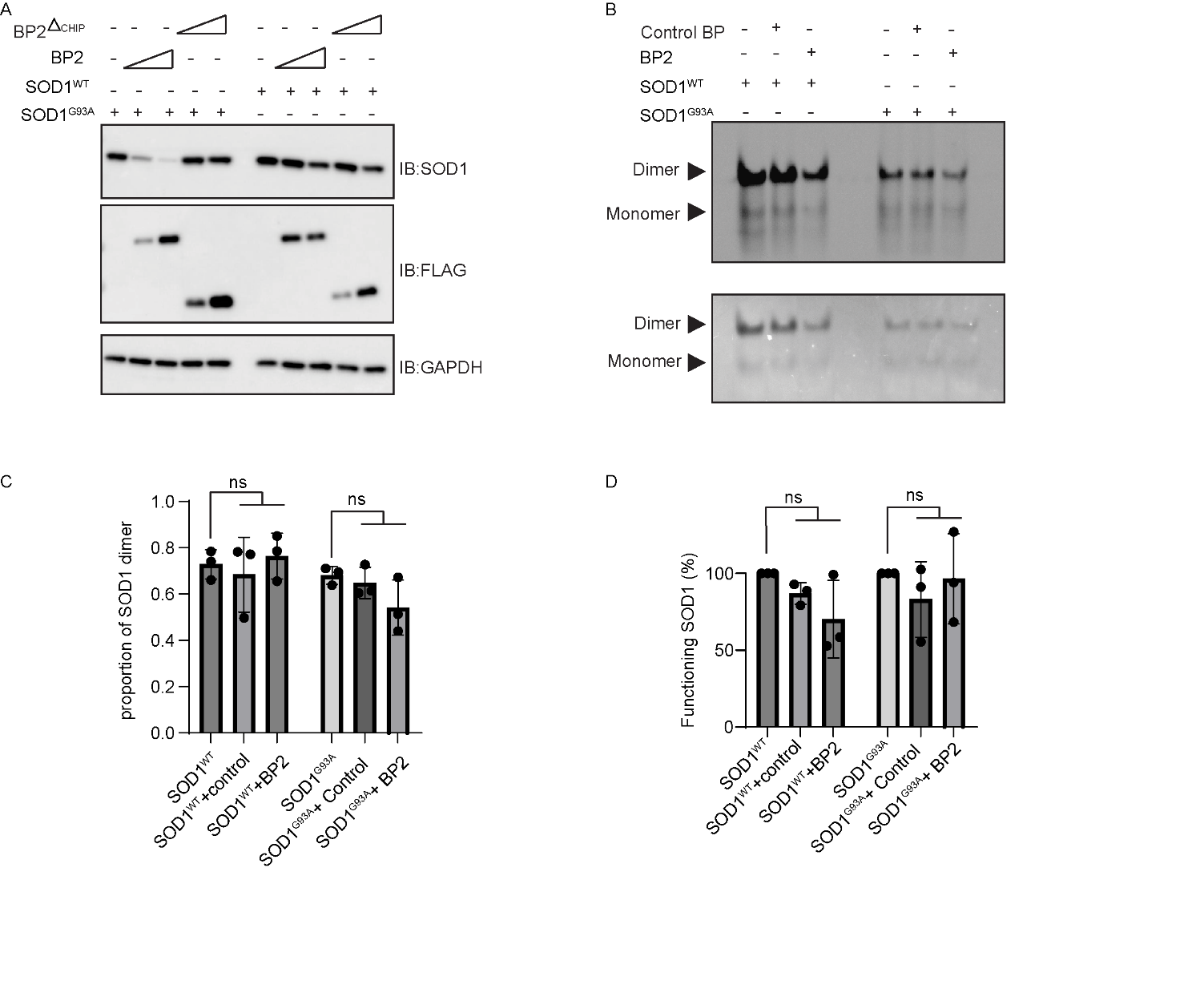


# Supplementary Figure 4

**.** **BioPROTAC 2 is sensitive to SOD1 folding**. **(A)** Levels of SOD1^WT^ and SOD1^G93A^ in HEK293 cells transfected with increasing amounts of BP2 or BP2^ΔCHIP^. **(B)** Native-PAGE of lysates from cells co-transfected with SOD1^WT^ or SOD1^G93A^ and Control or BP2 showing dimers and monomers (top) and in gel zymography of the same gels showing the relative activity of each species. **(C)** Quantification of fluorescence signal from native-PAGE of the proportion of SOD1–EGFP dimer, **(D)** Quantification of active dimeric SOD1 from in-gel zymography, normalised to EGFP fluorescence signal. For all graphs, bars represent mean ± SEM. Statistical significance was determined using **(C)** ordinary one-way ANOVA paired with Tukey’s multiple comparisons test. All statistical values are shown in Supplementary Table 2. Blots are representative from at least 3 independent experiments. Raw data and exact P-values are shown in source data file.


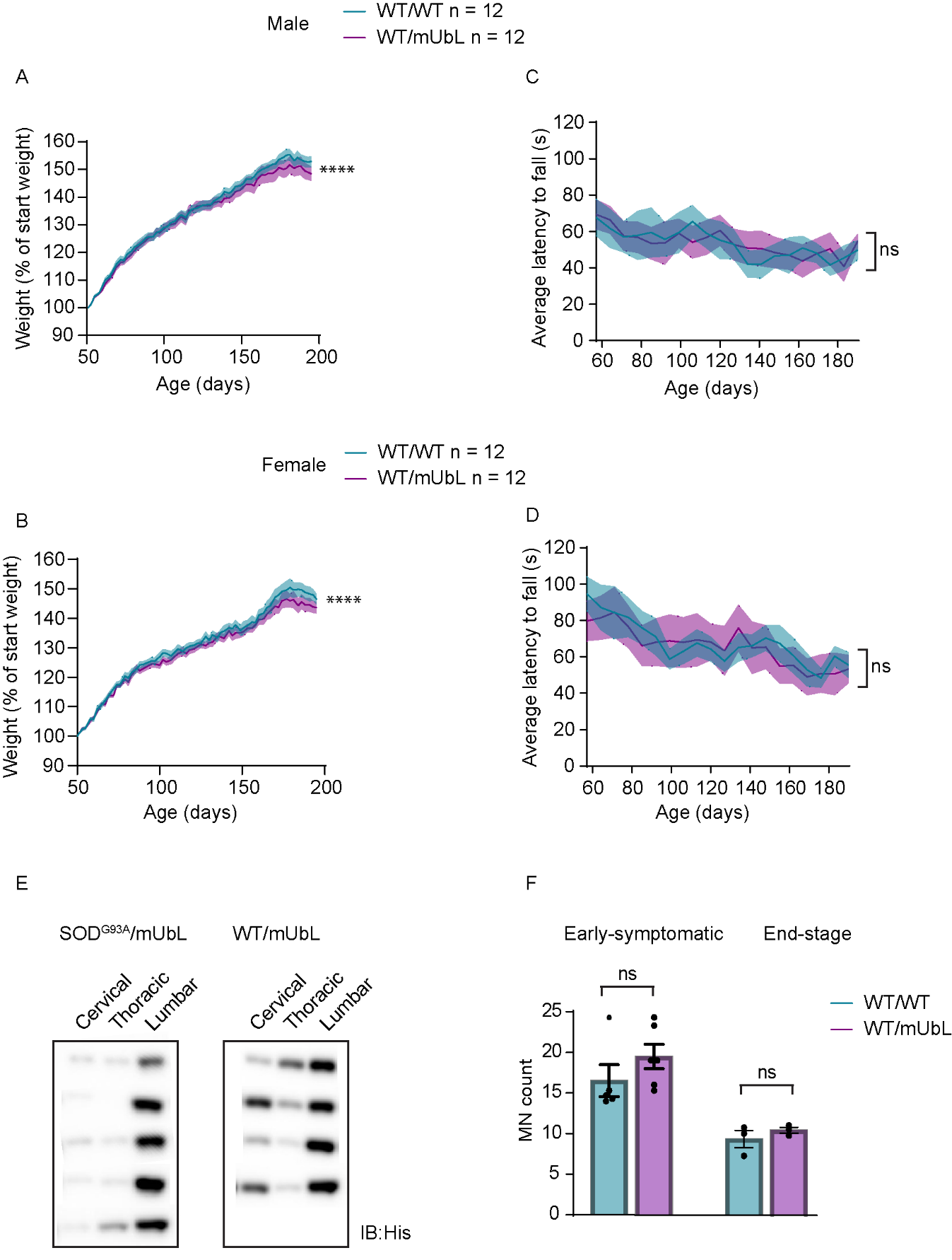


# Supplementary Figure 5

**. Phenotypic differences across the lifespan of non-transgenic WT/WT and WT/MisfoldUbL mice and expression of MisfoldUbL across spinal cord**  **(A-D)** Phenotypic data comparing WT/WT (n = 12 male and n = 12 female) and WT/MisfoldUbL control mice (n = 12 male and n = 12 female) are shown. Control groups were obtained as described in Figure 5D. Male and female mice were assessed for **(A and B)** weight gain and **(C and D)** motor function via latency to fall on the rotarod. Results represent mean ± SEM (shading) (**** *P* < 0.0001). **(E)** Immunoblot to assess relative expression of MisfoldUbL across the spinal cord in SOD1^G93A^/mUbL and WT/mUbL mice. **(F)** Motor neuron numbers in the ventral lumbar spinal cord of mice at early-symptomatic for SOD1 transgenic mice and 200 days old (post end-stage for SOD1 transgenic mice). For all graphs, bars represent mean ± SEM. Statistical significance was determined using **(A-D)** repeated measures one-way ANOVA paired with Tukey’s multiple comparisons test or **(F)** unpaired two-tailed t-tests. Raw data and exact P-values are shown in source data file.

# Supplementary Table 1

**.** Antibodies used to investigate Development of a targeted BioPROTAC degrader selective for misfolded SOD1

| Antibody | Conjugate | Antibody type | Company | Catalogue number | Dilution | Clonality | Application |
| --- | --- | --- | --- | --- | --- | --- | --- |
| anti-6X His | - | primary | Abcam | ab18184 | 1:1,000 | monoclonal | immunocytochemistry, co-immunoprecipitation |
| anti-mouse IgG | Alexa Fluor 647 | secondary | Abcam | ab150115 | 1:1,000 | polyclonal | immunocytochemistry |
| anti-FLAG | - | primary | Sigma | F1804 | 1:1,000 | monoclonal | immunoblotting |
| anti-6X His | - | primary | Abcam | ab9108 | 1:1,000 | polyclonal | immunoblotting |
| anti-SOD1 | - | primary | Abcam | ab13498 | 1:5,000 | polyclonal | immunoblotting |
| anti-GAPDH | - | primary | Sigma | G8795 | 1:5,000 | monoclonal | immunoblotting |
| anti-Hsp70 | - | primary | Abcam | ab47455 | 1:1,000 | monoclonal | immunoblotting |
| anti-mouse IgG | HRP | secondary | Agilent | P044701-2 | 1:1,000 | polyclonal | immunoblotting |
| anti-rabbit IgG | HRP | secondary | Agilent | P044801-2 | 1:1,000 | polyclonal | immunoblotting |
| anti-synaptophysin | - | primary | Abcam | ab32594 | 1:800 | polyclonal | NMJ immunostaining |
| anti-neurofilament heavy polypeptide | - | primary | Abcam | ab8135 | 1:1,000 | polyclonal | NMJ immunostaining |
| anti-rabbit IgG | Alexa Fluor 647 | secondary | Abcam | ab150079 | 1:500 | polyclonal | NMJ immunostaining |
| alpha-bungarotoxin | Alexa Fluor 488 | secondary | Thermo Fisher Scientific | B13422 | 1:2,000 | - | NMJ immunostaining |

GAPDH, glyceraldehyde 3-phosphate dehydrogenase; His, histidine; HRP, horseradish peroxidase; Hsp70, heat shock protein 70; IgG, immunoglobulin G; NMJ, neuromuscular junction; SOD1, superoxide dismutase 1.

# Supplementary Table 2

**.** Descriptive statistics for Figure 2F, Figure 3D and Supplementary Figure 3A

| Figure and Panel | Sample | BP expression (RFU) | | | SOD1-EGFP expression (RFU) | | |
| --- | --- | --- | --- | --- | --- | --- | --- |
|  |  | ***n*** | **Mean** | **SEM** | ***n*** | **Mean** | **SEM** |
| Figure 2F | Control BP | 3445 | 1 | 0.012 | 3445 | 64.55 | 0.922 |
|  | BP 1 | 2420 | 1.322 | 0.018 | 2420 | 27.3 | 0.406 |
|  | BP 2 | 3429 | 0.905 | 0.009 | 2665 | 13.97 | 0.125 |
|  | BP 3 | 1867 | 8.452 | 0.192 | 1867 | 4.633 | 0.053 |
|  | BP 4 | 2665 | 3.722 | 0.048 | 3429 | 47.1 | 0.47 |
|  | BP 5 | 3169 | 2.126 | 0.027 | 3169 | 23.13 | 0.221 |
|  | BP 6 | 2830 | 1.002 | 0.013 | 2830 | 42.79 | 0.449 |
|  | BP 7 | 3304 | 1.279 | 0.014 | 3304 | 52.23 | 0.587 |
| Figure 3D | Control | 2565 | 2.311 | 0.007 | 2565 | 2.056 | 0.002 |
|  | CHIP | 3069 | 2.374 | 0.005 | 3069 | 1.821 | 0.002 |
|  | UBE4A | 2911 | 2.385 | 0.005 | 2911 | 1.853 | 0.002 |
|  | NEDD4L | 6408 | 1.757 | 0.002 | 6408 | 2.346 | 0.002 |
|  | UBR5 | 6216 | 1.766 | 0.002 | 6216 | 2.367 | 0.002 |
|  | Parkin | 2945 | 2.372 | 0.006 | 2945 | 1.876 | 0.002 |
|  | UBOX5 | 5593 | 1.822 | 0.002 | 5593 | 2.381 | 0.002 |
|  | βTrCP | 3044 | 1.85 | 0.006 | 3044 | 2.175 | 0.003 |
|  | RNF4 | 3204 | 2.333 | 0.005 | 3204 | 1.828 | 0.002 |
| Supp. Figure 3A | Control | 2477 | 2.31 | 0.007 | 2477 | 2.196 | 0.008 |
|  | CHIP | 2806 | 2.379 | 0.005 | 5960 | 1.857 | 0.004 |
|  | UBE4A | 3073 | 2.384 | 0.005 | 3729 | 1.799 | 0.008 |
|  | NEDD4L | 6326 | 1.747 | 0.002 | 5901 | 1.915 | 0.005 |
|  | UBR5 | 5901 | 1.759 | 0.003 | 2943 | 1.99 | 0.006 |
|  | Parkin | 3051 | 2.357 | 0.005 | 6326 | 1.883 | 0.005 |
|  | UBOX5 | 5960 | 1.787 | 0.002 | 3073 | 2.065 | 0.006 |
|  | BTrCP | 3729 | 1.819 | 0.005 | 2806 | 2.074 | 0.006 |
|  | RNF4 | 2943 | 2.148 | 0.007 | 6147 | 1.985 | 0.005 |

BP, BioPROTAC; βTrCP, beta-transducin repeat-containing protein; CHIP, C-terminus of Hsc70 interacting protein; N, sample size; NEDD4L, neural precursor cell expressed developmentally downregulated 4-like; RNF4, RING finger protein 4; RFU, relative fluorescence units; SEM, standard error of the mean; SOD1-EGFP, superoxide dismutase 1-enhanced green fluorescent protein; Supp., supplementary; UBE4A, ubiquitination factor E4A; UBOX5, U-Box domain containing 5; UBR5, ubiquitin protein ligase E3 component N-recognin 5.

**Uncropped western blots included in Supplementary Information**


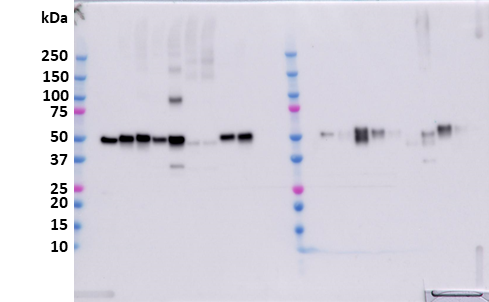

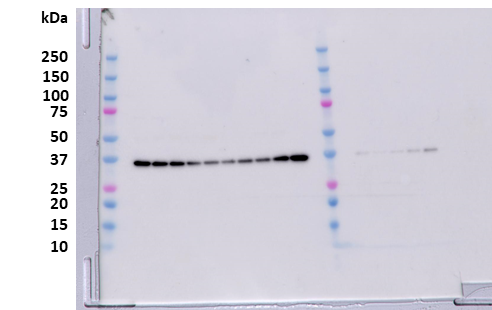


Uncropped images from Figure 1A.


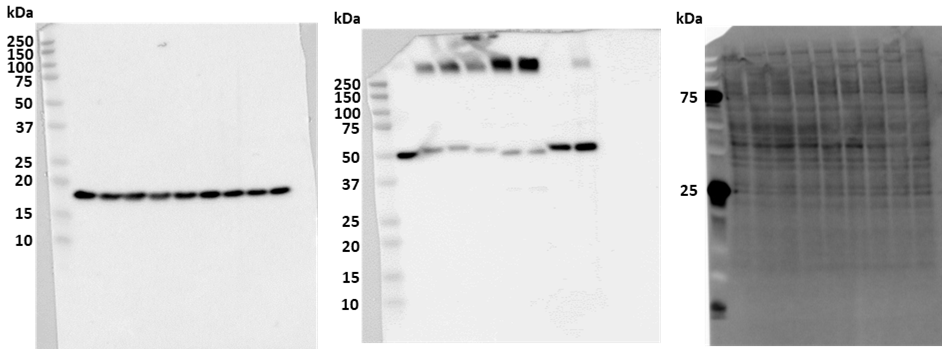


Uncropped images from Figure 1D.


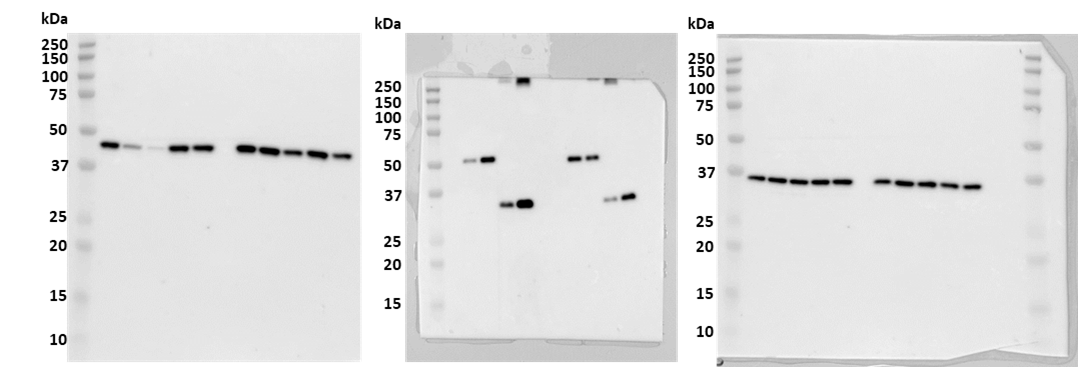


Uncropped images from Figure 4A.


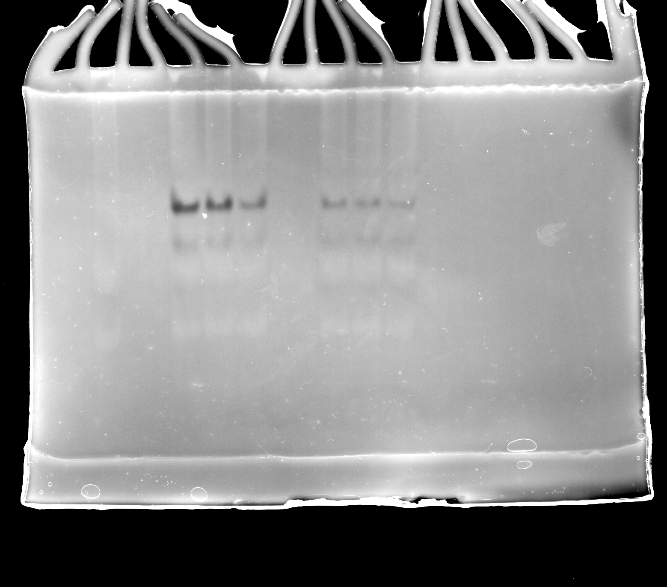

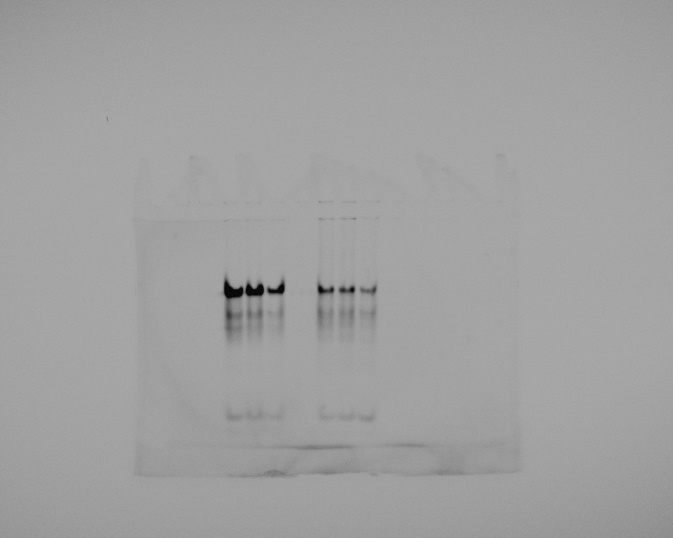
Uncropped images from Figure 4B


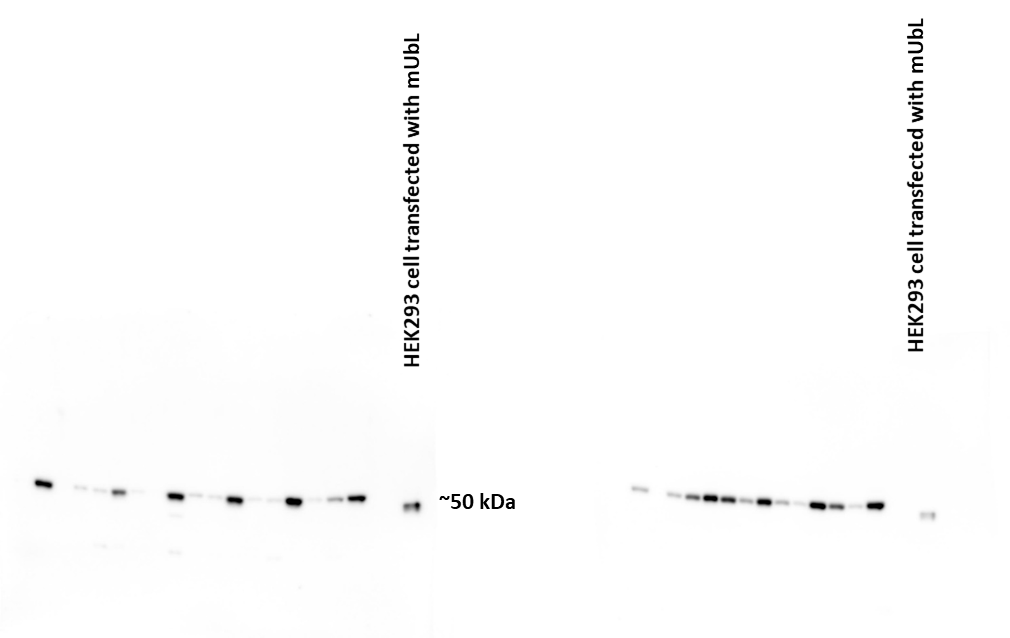


Uncropped images from Figure 5E
