# Supplementary material for: Development of a targeted BioPROTAC degrader selective for misfolded SOD1
Source: Nat Commun. 2025 Nov 10;16:9713. doi: 10.1038/s41467-025-65481-w (PMC12603324; doi:10.1038/s41467-025-65481-w)
Supplement: Supplementary file 2 — Description of Additional Supplementary Files [file 41467_2025_65481_MOESM2_ESM.docx]

**Description of Additional Supplementary Files**

# Supplementary Video 1

**.** SOD1^G93A^/mUbL mouse at 182 days and end-stage due to 20% weight loss criteria.

# Supplementary Video 2

**.** SOD1^G93A^/WT mouse at 174 days and end-stage due to reaching a neurological score of 4.
